# Considering scores between unrelated proteins in the search database improves profile comparison

**DOI:** 10.1186/1471-2105-10-399

**Published:** 2009-12-04

**Authors:** Ruslan I Sadreyev, Yong Wang, Nick V Grishin

**Affiliations:** 1 Howard Hughes Medical Institute, University of Texas Southwestern Medical Center, 5323 Harry Hines Blvd, Dallas, TX 75390-9050, USA; 2 Department of Biochemistry, University of Texas Southwestern Medical Center, 5323 Harry Hines Blvd, Dallas, TX 75390-9050, USA; 3 Biomedical Engineering Program, University of Texas Southwestern Medical Center, 5323 Harry Hines Blvd, Dallas, TX 75390-9050, USA; 4 The Human Genome Sequencing Center, Baylor College of Medicine, One Baylor Plaza, Houston, 77030, USA

## Abstract

**Background:**

Profile-based comparison of multiple sequence alignments is a powerful methodology for the detection remote protein sequence similarity, which is essential for the inference and analysis of protein structure, function, and evolution. Accurate estimation of statistical significance of detected profile similarities is essential for further development of this methodology. Here we analyze a novel approach to estimate the statistical significance of profile similarity: the explicit consideration of background score distributions for each database template (subject).

**Results:**

Using a simple scheme to combine and analytically approximate query- and subject-based distributions, we show that (i) inclusion of background distributions for the subjects increases the quality of homology detection; (ii) this increase is higher when the distributions are based on the scores to all known non-homologs of the subject rather than a small calibration subset of the database representatives; and (iii) these all known non-homolog distributions of scores for the subject make the dominant contribution to the improved performance: adding the calibration distribution of the query has a negligible additional effect.

**Conclusion:**

The construction of distributions based on the complete sets of non-homologs for each subject is particularly relevant in the setting of structure prediction where the database consists of proteins with solved 3D structure (PDB, SCOP, CATH, etc.) and therefore structural relationships between proteins are known. These results point to a potential new direction in the development of more powerful methods for remote homology detection.

## Background

The accuracy of detecting remote protein sequence relationships is essential for the inference and analysis of protein structure, function, and evolution. With the sample of solved structural folds growing close to the complete coverage of protein world [1], the ability to confidently detect a distant homolog with known 3D structure is becoming the major limiting factor in the fold prediction for any given protein sequence. Currently this ability is far from perfect, as once again highlighted by the recent Critical Assessment of Techniques for Protein Structure Prediction, CASP8 [2].

Comparing protein families, as profiles or hidden Markov models (HMMs) derived from multiple sequence alignments (MSA), rather than individual sequences, introduces information about the evolutionary constraints on sequence patterns dictated by protein structure and function, and therefore improves the quality of remote homology detection [3-11]. The similarity score of optimal profile-profile alignment is strongly influenced by residue composition, secondary structure (SS), and other features of the query and the template (subject) families. Depending on these properties, the same score value can be highly significant for one pair of profiles and marginal for another. Thus an important methodological step is the estimation of statistical significance of a similarity score, so that the most distant relationships are discriminated from spurious hits. This estimation is typically based on a background distribution inferred from the query's properties, which is generated from the scores for the random comparisons of unrelated proteins, either simulated [5,7,12,13] or real [4,9-11]. We have previously shown that improving the accuracy of the background distributions results in the increased detection quality of MSA comparison [14].

Although highly effective, this query-centered approach to the statistical significance cannot adequately reflect the uneven properties of different MSAs in the search database: subjects have different propensity to appear as a highly scored match when compared to an unrelated query. As adjustments to these propensities, multiple implicit schemes have been proposed that account for the subject's properties at the step of alignment score calculation, ranging from low-complexity filtering [15] to composition-based score rescaling [15] and composition-specific substitution matrices [16,17].

Here we consider an explicitly symmetrical approach to the background modeling at the step of estimating statistical significance, based on combining the background distributions for both query and subject. As a first step in this direction, we use a primitive scheme to combine query- and subject-based score distributions, and show that (i) inclusion of background distributions for the subject increases the quality of homology detection; (ii) this increase is higher when the distributions are based on the scores to all known non-homologs rather than a calibration subset of the database representatives; and (iii) these distributions make the dominant contribution to the improved performance: the removal of query-based calibration does not significantly deteriorate the performance.

## Results

Distributions of scores for the comparisons of a query to real database profiles (in contrast to profiles generated under a certain random model, e.g. shuffling of profile columns) are often used to estimate the statistical significance of a similarity between this query and any given profile. A major problem in the construction of these distributions is filtering out query homologs that should not be included in the assessment of the statistical background. As a solution to this problem, a current state-of-the-art HHsearch method [9], builds the background distributions based on a calibration database that contains a single protein representative from each structural fold and thus should not include more than one query homolog.

Although this approach provides individualized treatment of each query, it does not explicitly distinguish between various profiles in the search database, which also differ in their propensity to produce random high-scoring alignments with non-homologs. Here we consider a further development of this scheme, which involves the combination of individual background distributions for the query and each subject. Although the same calibration database can be used for both query and subjects, its composition is usually different from the search database: different folds have a different representation in the protein world, varying from a single tight sequence family to a plethora of divergent proteins as in Rossmann-type folds, TIM-barrels, imunnoglobulins, etc. As a way to preserve the structure of the search database in the background distributions, we consider constructing these distributions from the comparisons of the subject to all of its non-homologs in the search database. This approach is possible in the particular setting of structure prediction, where the database consists of proteins with solved 3D structure (PDB, SCOP, CATH, etc.), which in most cases allows for a straightforward assignment of their homology relationships.

As a method to produce profile-profile similarity scores, we use our recently developed scoring system implemented in PROCAIN for profile comparison [10]. In brief, PROCAIN score for the similarity between two MSA positions includes four terms: a standard measure for residue composition [7] combined with three additional measures for SS, amino acid conservation, and sequence motifs [10]. The resulting positional scores are subjected to composition-based rescaling [7,15] and used to construct optimal local Smith-Waterman alignment of the two profiles. Based on alignment scores for profile pairs in the testing set of distant SCOP representatives, we apply different schemes of estimating statistical significance (E-value) and compare the corresponding receiver operating characteristic (ROC) curves.

### Effect of considering background distributions for individual subjects

**Figure 1:**
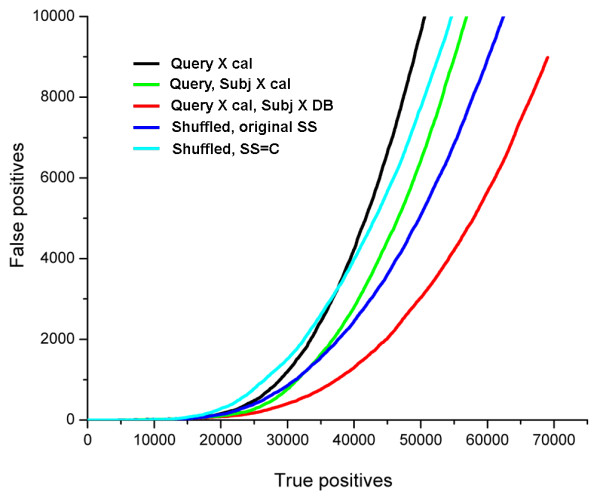
**Considering background distributions for individual subjects improves detection quality**. The standard approach based on the query calibration only (black ROC curve) is compared to the schemes that involve combining the query-based score distribution with individual subject-based distributions, produced by the comparison to the calibration database (green), to the set of all non-homologs in the search database (red), or, as controls, to the sets of randomized database profiles with shuffled positions: blue, profiles with the secondary structure assignment the same as in the real profiles; cyan, profiles with artificial secondary structure, 'coil' assigned to all positions. Combination of distributions produced by the comparison of query to the calibration database and of subject to the full set of its non-homologs results in the highest performance (red curve).

First, we assess the E-values based on two types of background score distributions: query calibration distribution alone and mixed with a background distribution for each individual subject. We consider several ways of modeling the subject distributions that involve the comparison of a subject to different profile sets representing unrelated proteins (Fig. 1). We use two sets of profiles for real protein families: the calibration database containing a single protein representative per fold (the same as used to calibrate query's background), and the full set of proteins from the search database that are classified as non-homologous to the subject. As a control, we use two sets of randomized profiles generated by random shuffling of positions in the profiles of the search database. These two artificial sets differ in the SS assigned to the profiles' positions, as a way to assess the contribution of SS to the background score modeling. In the first set, the shuffled profiles retain the same SS as the original real profiles; in the second set, all profile positions are uniformly assigned the coil SS (C).

As shown in Fig. 1, introduction of subject background distributions based on the calibration database improves the quality of similarity detection (green Vs black curve), with ROC increased from 0.21 to 0.24 on the set corresponding to the mean of 50 false positives per query (see also Table 1). The performance is further increased by including all non-homologs from the search database into the distribution (red curve), resulting in the ROC of 0.28 (Table 1). The distributions derived from the subject's comparison to the randomly shuffled profiles reduce detection quality to the levels comparable to or lower than the setting of query and subject comparison to the calibration subset. These distributions are significantly affected by the predicted SS of the database profiles: destruction of native SS patterns (cyan curve) leads to the performance inferior to that in the presence of original SS predictions (blue curve).

**Table 1:** ROC values

Scheme	ROC(2 10^4^)	ROC(2 10^5^)	ROC(745550)
Query × Cal	0.1020 ± 2 10^-4^	0.2099 ± 7 10^-5^	0.3135 ± 3 10^-5^

Query × Cal, Subj × DB	0.1427 ± 2 10^-4^	0.2819 ± 7 10^-5^	0.3990 ± 3 10^-5^

Shuffled DB, SS = C	0.1093 ± 2 10^-4^	0.2444 ± 7 10^-5^	0.3718 ± 3 10^-5^

Shuffled DB, original SS	0.1248 ± 2 10^-4^	0.2688 ± 7 10^-5^	0.3983 ± 3 10^-5^

Query, Subj × Cal	0.1146 ± 2 10^-4^	0.2382 ± 7 10^-5^	0.3540 ± 3 10^-5^

Subj × Cal	0.1074 ± 2 10^-4^	0.2268 ± 7 10^-5^	0.3462 ± 3 10^-5^

Subj × DB	01437 ± 2 10^-4^	0.2808 ± 7 10^-5^	0.3959 ± 4 10^-5^

Query × Cal × 4, Subj × DB	0.1323 ± 2 10^-4^	0.2676 ± 7 10^-5^	0.3844 ± 3 10^-5^

Query × Cal × 2, Subj × DB	0.1384 ± 2 10^-4^	0.2767 ± 7 10^-5^	0.3944 ± 3 10^-5^

Query × DB	0.1417 ± 2 10^-4^	0.2690 ± 7 10^-5^	0.3711 ± 3 10^-5^

Query × DB, Subj × DB	0.1607 ± 2 10^-4^	0.3052 ± 7 10^-5^	0.4184 ± 3 10^-5^

**Figure 2:**
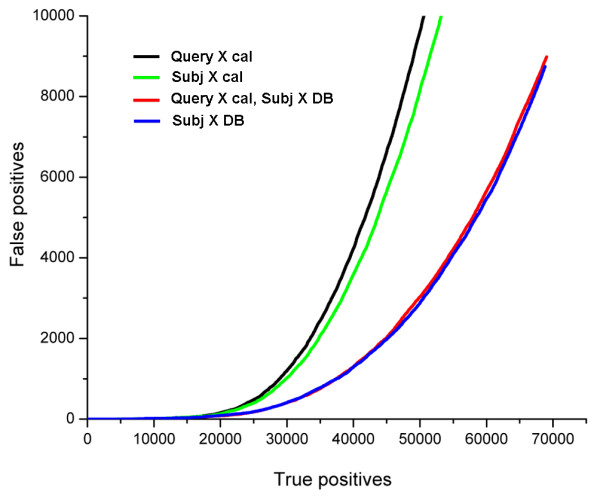
**Analysis of scores of subject to all database non-homologs has a dominant effect compared to query calibration**. The performance of the query calibration alone (black) and combined with subject calibration on the full database (red), compared to the subject calibration alone, using either the calibration database (green) or the full set of non-homologs (blue). Surprisingly, the latter scheme provides virtually the same detection quality regardless of whether query calibration scores are additionally considered (blue Vs red curve).

**Figure 3:**
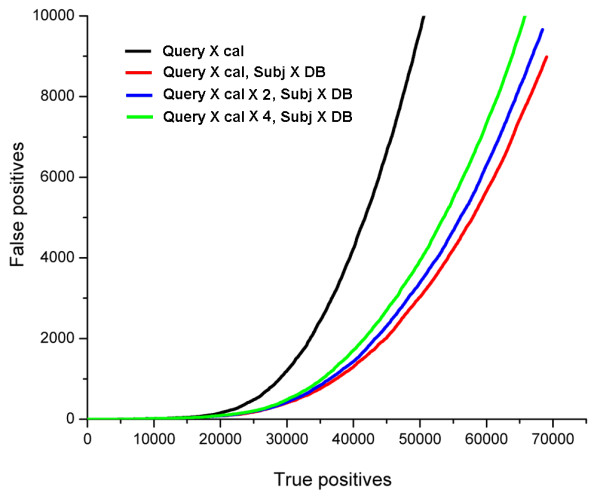
**Effect of the mixing ratio of query- and subject-based distributions**. The performance for the distributions produced by unweighted mixture of query- and subject-based scores (red curve) is compared to the schemes that introduce additional weighting, so that the samples of query and subject calibration scores have similar sizes. Increasing the weight of query calibration scores two-fold (blue) or four-fold (green) leads to a monotonic decrease of detection quality. Black, ROC curve for the query calibration alone.

**Figure 4:**
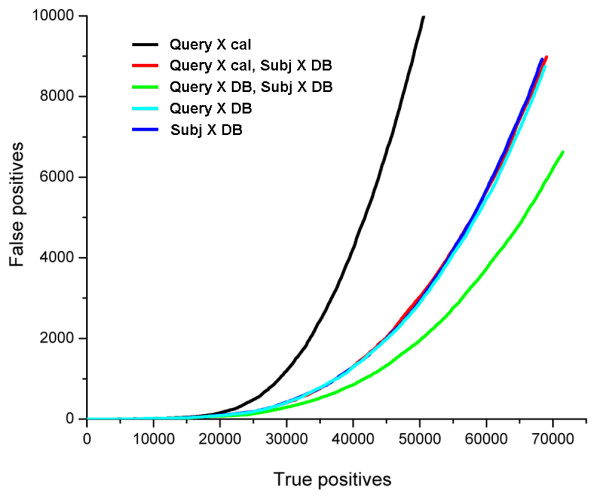
**Statistics based on the knowledge of all query's non-homologs: a possible hypothetical performance**. The detection quality that could potentially be achieved by using the distribution of query's scores to all non-homologs is compared to the performance of other schemes. ROC curve for the query-based distribution produced on the full set of non-homologs (cyan) is similar to that for the subject-based distribution produced on all non-homologs alone (blue) or mixed with query calibration distribution (red). This performance can be additionally improved by combining both query- and subject-based distributions generated on the full sets of their non-homologs (green). Black, ROC curve for the query calibration alone.

Receiver operating characteristics (ROC) for tested statistical schemes, calculated for different numbers of top false positives: the mean of 5 top false positives per query (2 10^4 ^false positives total); the mean of 50 top false positives per query (2 10^5 ^false positives total), and the point where PROCAIN retrieves half of all true positives in the dataset (745550 false positives total). The total number of true positives in the testing set is T = 474929. Statistical schemes are denoted the same way as in Fig. 1-4.

The separate evaluations of these statistical schemes on queries from different major SCOP classes are shown in Additional File 1: Fig. S1.

### Background distributions for subjects contribute more than background distribution for query

To assess the role of query calibration in our symmetrized scheme, we compare the method's performance in the settings with background distributions for the subjects used alone and mixed with the calibration distribution for the query. Fig. 2 shows ROC plots produced by query calibration alone (black) and combined with subject comparison to all non-homologs (red), compared to two settings with no consideration of query's background distribution: with E-values based only on the scores for the subject against the calibration database (green) or the full set of all non-homologs (blue).

Calibration of subject alone on the set of fold representatives leads to slightly better performance than calibration of query (Fig. 2), with the ROC values of 0.107 Vs 0.102 (Table 1). Using score distributions of subject against all non-homologs significantly increases detection quality (ROC = 0.28, Table 1). Surprisingly, this quality stays virtually the same when query calibration distribution is mixed in (Fig. 2, ROC value of 0.2819 ± 7 10^-5 ^Vs 0.2808 ± 7 10^-5^). This result suggests that the knowledge of all subject's non-homologs is a dominant factor in the improvement of the statistical estimates. This dominance has a potential practical implication: when homology relationships between database entries are defined, the calibration of query may become unnecessary since it does not contribute to the detection accuracy.

The separate evaluations of these statistical schemes on queries from different major SCOP classes are shown in Additional File 1: Fig. S2.

A possible explanation of this relatively weak influence of query calibration on the performance might be the difference in the sizes of score samples produced by the comparison to the calibration database (~1000 fold representatives) and all defined non-homologs in the full database (~4000 entries). In our original implementation, the scores from these two sources are mixed without additional weighting, therefore the contribution of the query calibration distribution is several fold smaller than the contribution of subject's distribution. As a control, we increase the weight of the query calibration in the mixture and observe the resulting performance (Fig. 3). Interestingly, increasing the weight of the query-based distribution up to the same level as the subject-based distribution (approximately four-fold) does not improve detection quality; in fact, the performance gradually deteriorates when the mixing ratio is changed (Fig. 3, Table 1).

The separate evaluations of these statistical schemes on queries from different major SCOP classes are shown in Additional File 1: Fig. S3.

### Effects of statistics based on the knowledge of all query's non-homologs

Since the use of the full set of subject's non-homologs has a significant advantage over the use of the calibration subset (Fig. 2), it is interesting to assess the performance in the hypothetical situation when the query calibration is also based on the full set of its non-homologs in the search database. In this setting, query-based background distributions and score averages (see Methods) are based on the query's non-homologs in the search database rather than on the calibration database. In Fig. 4, ROC plots for such query calibration alone (cyan) or together with subject calibration (green) are compared against plots for other settings. Similar to the result for subject-based distributions (Fig. 2), including all query non-homologs significantly improves the quality of statistical estimates, as compared to using only the calibration subset (Fig. 4), with ROC increasing from 0.21 to 0.27 (Table 1). Interestingly, the resulting performance is very similar to that for the individual subject-based distributions including all non-homologs of the subject. This performance is significantly improved by mixing both query- and subject-based distributions of scores against respective full non-homolog sets (Fig. 4), with ROC reaching the value of 0.31 (Table 1). These results suggest that considering all non-homologs in the background distributions on either query or subject side has approximately the same effect, and that combining these effects can contribute to the performance in an additive way.

The separate evaluations of these statistical schemes on queries from different major SCOP classes are shown in Additional File 1: Fig. S4.

## Discussion

Using MSA rather than single sequences for homology detection provides stronger similarity signals: patterns of residue usage at individual positions, conserved motifs, interdependence of residue content at different positions and other information help detecting remote relationships in the cases where individual sequences diverged beyond recognition. However, these signals create new challenges for the discrimination between MSA similarities caused by homology and produced by chance, for example by a local structural similarity involving a few SS elements in globally different proteins.

The calculation of profile-profile similarity scores in PROCAIN already includes implicit consideration of both query and subject properties [7,10] in a fashion similar to the composition-based statistic implemented in PSI-BLAST [15]. Specifically, the scale of all scores between individual positions of query and subject is forced to a standard level, which allows for using universal gap penalties and makes the scores of optimal profile-profile alignments compatible for different profile pairs. In PROCAIN's predecessor, COMPASS [7], these rescaled scores are used directly in the calculation of E-values according to Karlin-Altschul formula [18] with pre-computed parameters of statistical distributions derived from extensive upfront simulations. However, while developing PROCAIN, we found that deriving distribution parameters from the comparisons of real unrelated proteins leads to a better detection accuracy [10].

Here we analyze a novel approach to estimating statistical significance of similarity between MSAs, based on the explicit consideration of background distributions for both query and subject. In order to conduct a proof-of-principle study that would not rely on a specific statistical treatment of the data, we use a primitive mixing of PROCAIN scores and simple EVD approximations of the distributions, rather than more elaborate statistical formalisms. We find that (i) the quality of estimation of statistical significance for a given similarity score improves by incorporating information about statistical properties of the subject, and (ii) this improvement significantly increases when the full set of subject's non-homologs, rather than a subset of fold representatives, is used to infer the background distribution.

Our most unexpected finding is that the improvement in the performance is dominated by using the score distributions for all non-homologs of the subject, and that considering the calibration distribution of the query has almost no additional effect (Fig. 2). Conceptually, there are several potential sources of this improvement. First, detection quality may increase simply due to individualized treatment of each subject, as opposed to using query-based distribution in all comparisons. The second source is the combination of query and subject distributions resulting in better modeling of statistics on both sides of comparison. The third source is the more representative sampling in the construction of background distributions, given the knowledge of all actual non-homologs of the subject in the database. Based on our results, we can exclude the first source. Similarity in performance of 'subject-only' and 'query-only' distributions derived from the full set of non-homologs (Fig. 4), as well as from the calibration set (Fig. 2) suggests that individualized treatment of subjects alone cannot provide a dramatic increase in detection quality. According to the evaluations shown in Fig. 1 and 4, the largest source of improvement is the inclusion of the full set of non-homologs in the background modeling. This full sampling, normally impossible for the query, can be achieved for the subjects when relationships within the database are known. Combining subject- and query-based distributions, derived either from the calibration set or the full sets of non-homologs, makes an additional contribution to the improvement of performance.

The presented results suggest a potentially important approach to increasing the quality of remote homology detection. Effective implementation of this approach in new computational methods will require additional research in at least two areas: (i) fuller usage of the information about homology relations in the search database, for example, consideration of scores for homologs; and (ii) more detailed and accurate statistical treatment of mixed background score distributions.

## Conclusion

We present and analyze a novel approach to the estimation of statistical significance of profile-profile similarities, based on explicit consideration of both query and subject background score distributions. This approach provides a higher quality of homology detection than query calibration alone. A significant additional increase can be achieved by using the knowledge of actual homology relationships between subjects in the search database, which allows for a more representative sampling of statistical background for each subject. The presented results can serve as a basis for the development of more powerful methods for remote similarity detection.

## Methods

### Search and calibration databases

As a search database, we use the set of 4147 PSI-BLAST MSAs of homologs for SCOP domain representatives with less than 20% sequence identity that was constructed and extensively used as a part of a previously described benchmarking system [19]. MSAs for SCOP 1.69 domains were generated from homologs detected after up to 8 PSI-BLAST iterations with default parameters, combined with additional processing to remove fragments and eliminate obvious misalignments, followed by SS prediction by psipred [20]. The calibration database includes 935 profiles from the search database, chosen as a single representative per SCOP fold, the same domain set as used for query HMM calibration by HHSearch [9]. The lists of SCOP domains included in the search database and the calibration set are given in Additional Files 2 and 3, respectively.

### PROCAIN similarity scores

To produce profile similarity scores, PROCAIN [10] complements the standard measure for positional similarity of amino acid content with the measures for structure- and function-related patterns revealed by MSA: similarity in SS, amino acid conservation, and MSA motifs. For every database profile *A *we calculate the set of similarity scores against all non-homologous profiles and find the mean value of this set, <s>_A_. Then we process this set by subtracting the mean score of the counterpart profile *B *from each score s_AB _between *A *and *B*: *s'*_AB _= *s*_AB _- <s>_B_. The resulting distribution of scores {*s'*_AB_} for profile *A *is stored. In a similar fashion, for every profile *C *in the calibration database, we pre-compute the set of similarity scores {*s*_CA_} against entries of the searching database and then calculate the mean value of this set, <s>_C_. When query profile *Q *is compared to profiles in the calibration database; the mean score of each profile *C *is subtracted from its similarity score to the query s_QC_: *s'*_QC _= *s*_QC _- <s>_C_: During the actual search, when query *Q *is compared to profile *A *in the searching database, the distributions of adjusted calibration scores for the query, {*s'*_QC_}, and for the subject, {*s'*_AB_}, are analyzed and combined according to the scheme being evaluated. The resulting distribution is fitted with EVD to estimate EVD parameters *k *and λ, which are then used in Karlin-Altschul formula to calculate E-value: *E *= *kmne*^-λ*S*^, where *m *and *n *are effective lengths of the two profiles and *S *= *s*_QA _- 0.5(<s>_QC _+ <s>_A_) is the adjusted score for query against the database profile *A *[10]. The latter formula corresponds to the average of two scores corresponding to the two mixed distributions: *s'*_QA _= *s*_QA _- <s>_QC_, i.e. the score adjusted on the query side by the mean of query's calibration scores, and *s'*_AQ _= *s*_QA _- <s>_A_, i.e. the score adjusted on the subject side by the mean of subject's scores to its non-homologs.

In the setting when the calibration database is substituted by all query's non-homologs in the search database (Fig. 4), the query-based background distributions are generated on these non-homologs, and the adjusted score for query vs subject is calculated as *S *= *s*_QA _- 0.5(<s>_Q _+ <s>_A_), where <s>_Q _is the query's mean score against all non-homologous profiles.

### Evaluation of homology detection quality

To assess the detection quality, we build receiver operating characteristic (ROC) curves based on the results of searches use in the database of ~4000 SCOP representatives selected at 20% sequence identity of structure-based alignment described above. True and false positive definition combines expert assignments of superfamilies by SCOP and our automated SVM classifier based on multiple scores for sequence and structure similarity of the two proteins, applied as previously described [19]. These homology assignments reduce the number domain relationships classified in SCOP as undefined when the two domains share a SCOP fold but belong to different superfamilies. In brief, we constructed a classifier that combines multiple sequence- and structure- based similarity scores and trained it on a SCOP subset of 1000 pairs that belong to different SCOP classes and are labeled as negative for SVM training, and 1000 pairs that belong to the same SCOP superfamilies and are labeled positive. The five most dominant features of the resulting classifier are as follows: DALI Z-score, FAST score, coverage of FAST alignment, GDT_TS of TM alignment, and BLOSUM score of DALI alignment [19].

The resulting SVM makes a binary classification of domain pairs into the categories of similar and dissimilar. However, there is a number of domain pairs that share short regions of similarity but are poor global structural templates for each other (for example, Rossmann-type folds *vs*. TIM barrels). Forcing such cases to either of the two categories might bias the evaluation protocol. Therefore, following others, we use the third category of 'undefined' relations and establish the corresponding lower and higher thresholds of SVM score to define the three areas: dissimilar, unknown and similar [19], with unknowns comprising ~10% of all pairs [19].

Two proteins are classified as similar if they share a SCOP superfamily or have a high SVM score. They are classified dissimilar if do not share a superfamily and have a low SVM score. Relationships between domains from different superfamilies with intermediate SVM score are classified as unknown. We successfully tested and applied the resulting classification to the evaluation of various methods for remote homology detection [7,10].

ROC values and their error estimates are calculated as described in [15]: for the top *n *false positives ROC_*n *_= (1/*nT*)Σ_i = 1_^*n*^*t*_*i*_, where *t*_*i *_is the number of true positives that were ranked ahead the *i*th false positive in the list, and *T *= 474929 is the total number of true positives in the dataset.

### Randomly shuffled profiles

As negative controls, we build subject-based score distributions from the comparison of the subject to the set of randomized profiles produced by shuffling positions of original database profiles. To assess the contribution of predicted SS to the accuracy of the statistics, we compare the performance with the original SS assignments preserved in the randomized profiles to the performance with the artificial uniform coil SS assigned to all positions.

## Authors' contributions

RS generated the data (statistical schemes on shuffled profile databases, performance with known query homologs, varying distribution weights, ROC values, and performance on different SCOP classes), analyzed results, and drafted the manuscript. YW conceived of the original idea of using subject-based distributions and their importance for statistical estimates, as implemented it in the PROCAIN method, and generated part of the data (evaluation of different statistical modes of PROCAIN). NG conceived of the study and participated in its design and coordination. All authors read and approved the final manuscript.

## Supplementary Material

Additional file 1 **Performance of statistical schemes on different SCOP classes**. Supplementary Figures 1-4 (MSWord file) with ROC plots on separate SCOP classes. Click here for file

Additional file 2 **SCOP domains included in the search database**. List of SCOP domain representatives included in the search database (text file). Click here for file

Additional file 3 **SCOP domains included in the calibration database**. List of SCOP domain representatives included in the calibration database (text file). Click here for file
