# Supplementary material for: Considering scores between unrelated proteins in the search database improves profile comparison
Source: BMC Bioinformatics. 2009 Dec 4;10:399. doi: 10.1186/1471-2105-10-399 (PMC3087343; doi:10.1186/1471-2105-10-399)
Supplement: Additional file 1 — Performance of statistical schemes on different SCOP classes. Supplementary Figures 1-4 (MSWord file) with ROC plots on separate SCOP classes. [file 1471-2105-10-399-S1.DOC]

Considering scores between unrelated proteins in the search database improves profile comparison

Ruslan I. Sadreyev, Yong Wang, and Nick V. Grishin

**Supplementary Data**

**Figure S1**


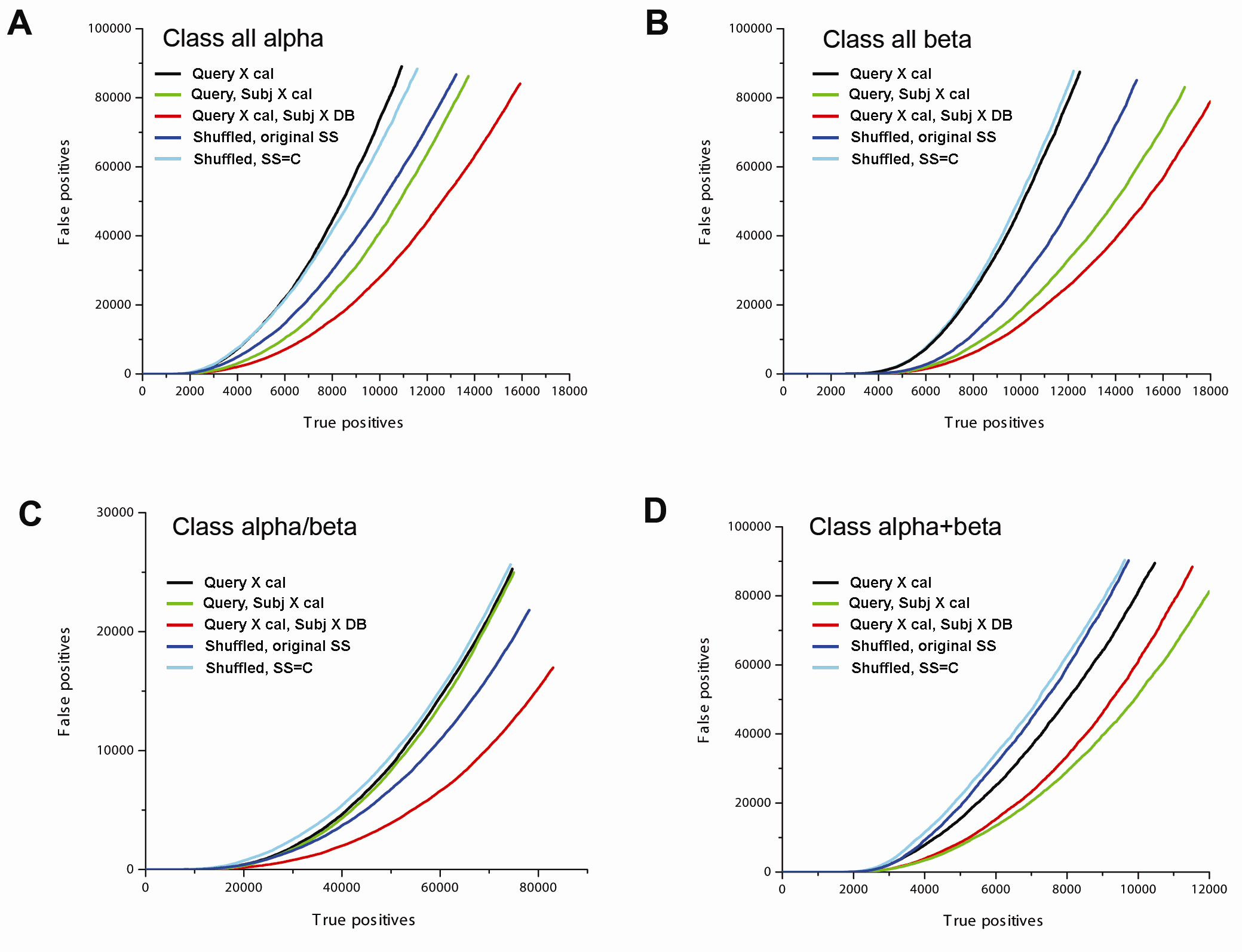


**Figure S1. Considering background distributions for individual subjects improves detection quality: ROC curves for queries of different SCOP classes.** The standard approach based on the query calibration only (black) is compared to the schemes that involve combining the query-based score distribution with individual subject-based distributions, produced by the comparison to the calibration database (green), to the set of all non-homologs in the search database (red), or, as controls, to the sets of randomized database profiles with shuffled positions: blue, profiles with the secondary structure assignment the same as in the real profiles; cyan, profiles with artificial secondary structure, ‘coil’ assigned to all positions.

**Figure S2**


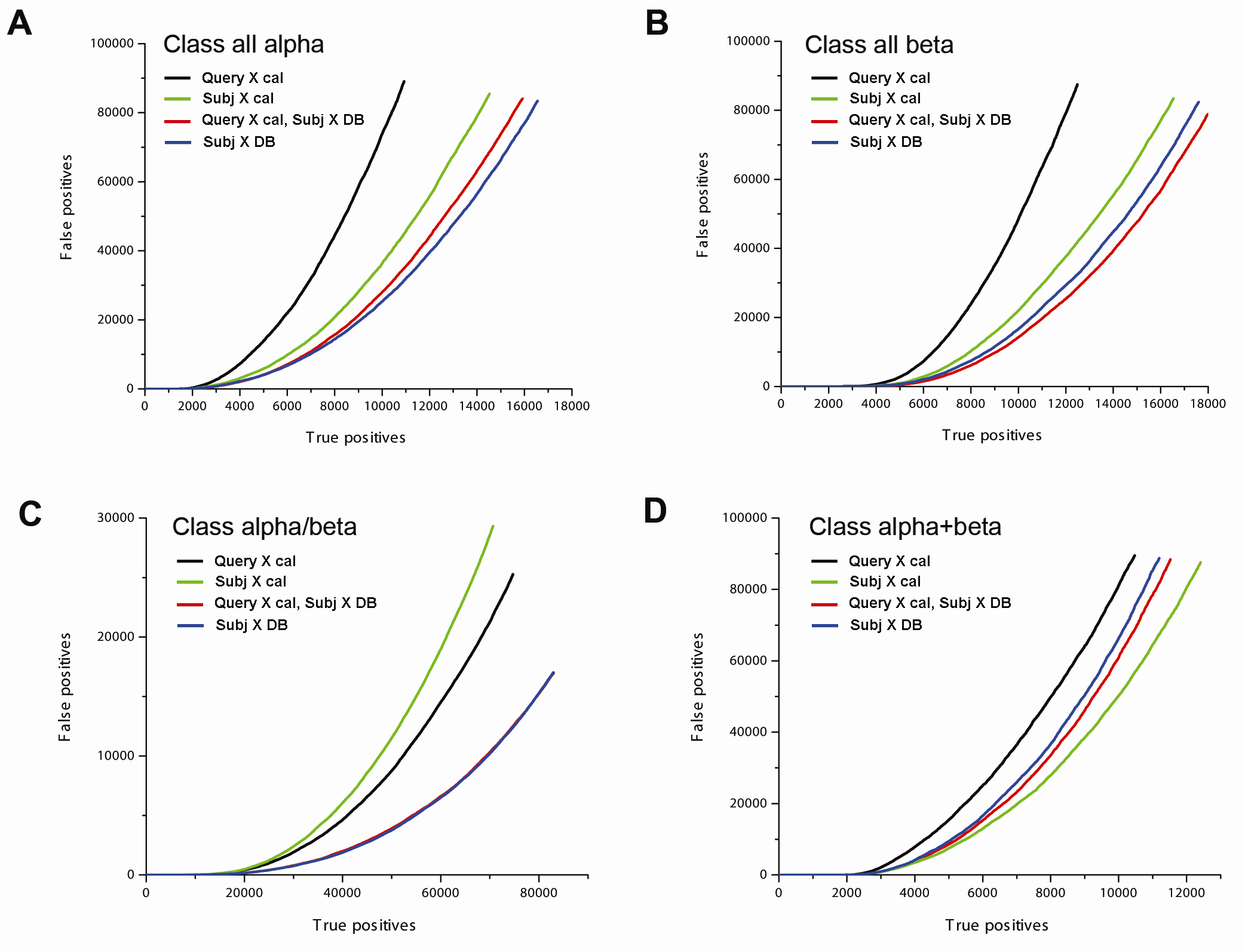


**Figure S2. Analysis of scores of subject to all database non-homologs has a dominant effect compared to query calibration: ROC curves for queries of different SCOP classes.** The performance of the query calibration alone (black) and combined with subject calibration on the full database (red), compared to the subject calibration alone, using either the calibration database (green) or the full set of non-homologs (blue).

**Figure S3**


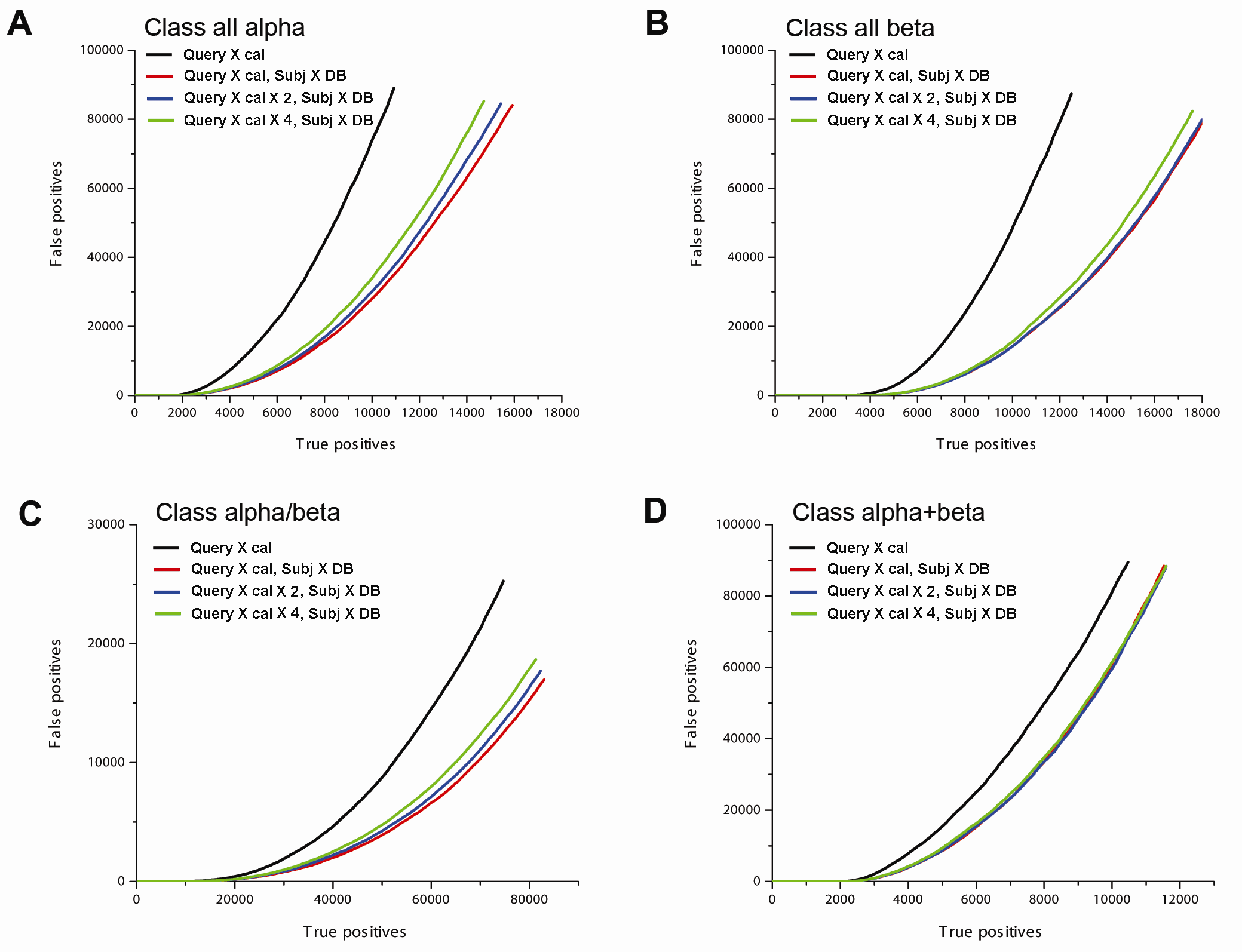


**Figure S3. Effect of the mixing ratio of query- and subject-based distributions: ROC curves for queries of different SCOP classes.** The performance for the distributions produced by unweighted mixture of query- and subject-based scores (red) is compared to the schemes that introduce additional weighting, so that the samples of query and subject calibration scores have similar sizes. The weight of query calibration scores is artificially increased two-fold (blue) or four-fold (green). Black, ROC curve for the query calibration alone.

**Figure S4**


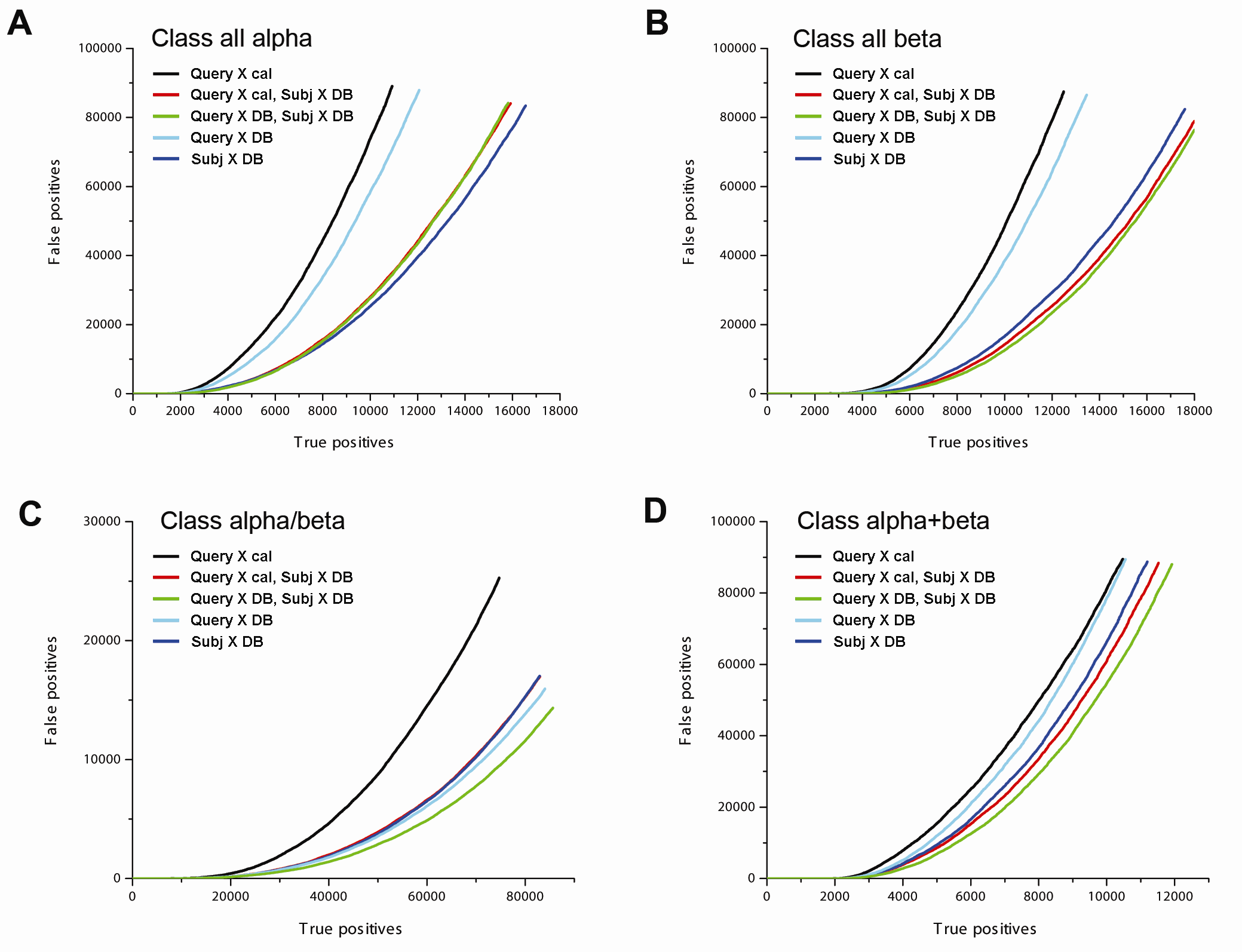


**Figure S4. Statistics based on the knowledge of all query’s non-homologs: a possible hypothetical performance: ROC curves for queries of different SCOP classes.** The detection quality that could potentially be achieved by using the distribution of query’s scores to all non-homologs is compared to the performance of other schemes. ROC curve for the query-based distribution produced on the full set of non-homologs (cyan) is shown together with the curves for the subject-based distribution produced on all non-homologs alone (blue) or mixed with query calibration distribution (red). Green, combining both query- and subject-based distributions generated on the full sets of their non-homologs. Black, query calibration alone.
